# Nicotine protects rat hypoglossal motoneurons from excitotoxic death via downregulation of connexin 36

**DOI:** 10.1038/cddis.2017.232

**Published:** 2017-06-15

**Authors:** Silvia Corsini, Maria Tortora, Rossana Rauti, Andrea Nistri

**Affiliations:** 1 Department of Neuroscience, International School for Advanced Studies (SISSA), Trieste, Italy

## Abstract

Motoneuron disease including amyotrophic lateral sclerosis may be due, at an early stage, to deficit in the extracellular clearance of the excitatory transmitter glutamate. A model of glutamate-mediated excitotoxic cell death based on pharmacological inhibition of its uptake was used to investigate how activation of neuronal nicotinic receptors by nicotine may protect motoneurons. Hypoglossal motoneurons (HMs) in neonatal rat brainstem slices were exposed to the glutamate uptake blocker DL-threo-*β*-benzyloxyaspartate (TBOA) that evoked large Ca^2+^ transients time locked among nearby HMs, whose number fell by about 30% 4 h later. As nicotine or the gap junction blocker carbenoxolone suppressed bursting, we studied connexin 36 (Cx36), which constitutes gap junctions in neurons and found it largely expressed by HMs. Cx36 was downregulated when nicotine or carbenoxolone was co-applied with TBOA. Expression of Cx36 was preferentially observed in cytosolic rather than membrane fractions after nicotine and TBOA, suggesting protein redistribution with no change in synthesis. Nicotine raised the expression of heat shock protein 70 (Hsp70), a protective factor that binds the apoptotic-inducing factor (AIF) whose nuclear translocation is a cause of cell death. TBOA increased intracellular AIF, an effect blocked by nicotine. These results indicate that activation of neuronal nicotinic receptors is an early tool for protecting motoneurons from excitotoxicity and that this process is carried out via the combined decrease in Cx36 activity, overexpression of Hsp70 and fall in AIF translocation. Thus, retarding or inhibiting HM death may be experimentally achieved by targeting one of these processes leading to motoneuron death.

Amyotrophic lateral sclerosis (ALS) is characterized by motoneuron death in the brainstem and spinal cord. The etiopathology seems to involve a complex interplay among pathogenic factors such as glutamate-mediated excitotoxicity, reactive oxygen species (ROS) and mitochondrial dysfunction.^1^ The brainstem nucleus hypoglossus is often affected early, thus leading to dysarthria and dysphagia.^2^ Hypoglossal motoneurons (HMs) are very vulnerable because of their high basal intracellular Ca^2+^ref. (3) expression of Ca^2+^ permeable AMPA receptors^4^ and low levels of glutamate transporters.^5^ In ALS, these properties are exacerbated by impaired glutamate transport^5, 6^ and increased level of glutamate in cerebrospinal fluid^7^ making excitotoxicity an important causative candidate. Motoneuron death shows patchy distribution and slow progression^8^ associated with muscle weakness and fasciculations.^9^

In the past few years, our laboratory has developed a simple *in vitro* model of excitotoxic stress applied to rat HMs.^10, 11, 12^ It consists of mimicking the pathological process of motoneuron hyperexcitability and excitotoxicity in the nucleus hypoglossus through pharmacological inhibition of glutamate uptake with DL-threo-*β*-benzyloxyaspartate (TBOA). Salient characteristics of this model are generation of electrical bursting among HMs interconnected via gap junctions^12^ and slow onset of cell death^11^ via production of ROS and subsequent mitochondrial energy deficit in a subset of motoneurons.^13, 14^ One essential property of HMs is their ability to generate 'group bursting' dependent on the membrane expression of gap junctions^15, 16^ made by connexins, of which connexin 36 (Cx36) is highly represented in the nervous system.^17, 18^ Gap junctions may also be due to pannexins, particularly pannexin 1 (Panx1), which share, with connexins, similar topology, permeability and gating.^18, 19^ In case of cell stress, expression of connexins and pannexins may increase the probability of damage diffusion and cell death.^17^

We recently observed that the neuronal acetylcholine receptor (nAChR) agonist nicotine prevents HM dysfunction and associated cell death.^13, 14^ Although these effects are dependent on nAChR activation,^13^ it seems likely that nicotine was simply a trigger for intracellular and network events contrasting excitotoxicity. Within this framework, on rat brainstem motoneurons nicotine induces early facilitation of glutamate release followed by depression together with enhancement of inhibitory neurotransmission.^20^ As a result of the complexity of the brainstem circuitry and its afferent projections, it is likely that the result of nicotine activity is the outcome of interplay among a variety of mechanisms. To better understand the action of nicotine, the present work investigated the functional topography of responsive neurons first and, subsequently, the processes of their modulation. One lead in this direction was provided by the report that in cultured endothelial cells nicotine downregulates connexins.^21, 22^ Thus, we wondered if a similar process could account for neuroprotection of HMs. To this aim, we studied how nicotine could modulate Ca^2+^ transients, Cx36 and Panx1, and compared it with the effects of the gap junction blocker carbenoxolone.^23^ In analogy with previous reports,^24, 25^ we used expression of heat shock protein 70 (Hsp70) and apoptosis-inducing factor (AIF) as indices of cell protection or death, respectively.

## Results

### Network distribution of Ca^2+^ signals induced by TBOA

**Figure 1:**
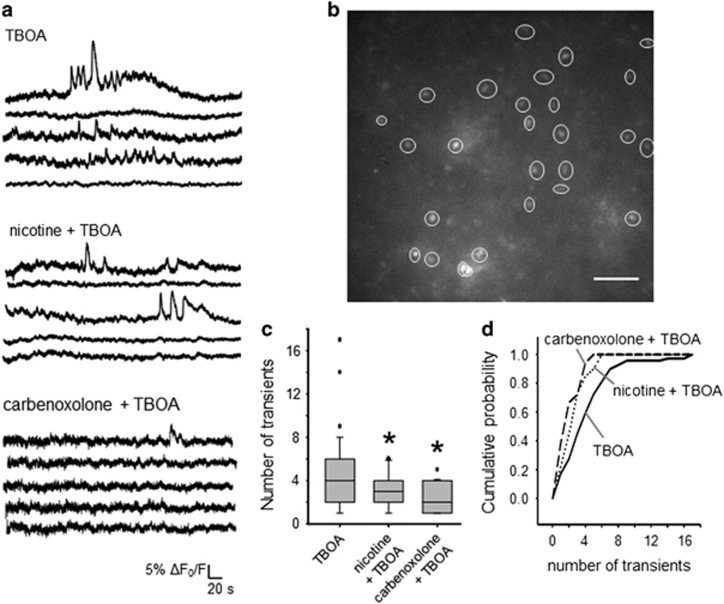
[Ca^2+^]_i_ transients evoked by TBOA. (**a**) Example of records of [Ca^2+^]_i_ changes simultaneously taken from five HMs in the same slice preparation in presence of TBOA (50 *μ*M; top), nicotine (10 *μ*M) + TBOA (middle), or carbenoxolone (200 *μ*M) + TBOA (bottom). (**b**) Example of slice preparation containing HMs. Each ROI indicates a different cell used for analysis. Bar=50 *μ*m. (**c**) Box plot of the number of transients evoked during the different treatment listed above; nicotine or carbenoxolone co-application with TBOA significantly reduces the number of the events (Kruskal–Wallis one-way ANOVA: *P=0.001; Dunn’s method: *P*<0.05 for TBOA *versus* nicotine + TBOA, and for TBOA *versus* carbenoxolone + TBOA; *n*: 69, TBOA; 40, nicotine + TBOA; 18, carbenoxolone + TBOA; *N*=5). (**d**) When TBOA is applied alone, the cumulative probability line (solid line) is significantly shifted to the right compared with nicotine + TBOA (dotted line) or carbenoxolone + TBOA (dashed line; Kolmogorov–Smirnov statistic test: *P*≤0.001 for TBOA *versus* nicotine + TBOA, and *P*≤0.001 for TBOA *versus* carbenoxolone + TBOA)

TBOA (50 *μ*M) induces bursting activity in about 50% of HMs^12, 13^ with large inward currents translated into strong intracellular Ca^2+^ transients.^26^ This study investigated [Ca^2+^]_i_ changes in the hypoglossal nucleus (Figures 1a, b and Supplementary Movies 1–3) to monitor excitation spread. Thus, we performed continuous imaging (10 min) of [Ca^2+^]_i_ transients as shown in Figure 1b and Supplementary Movies 1–3, in which about 30 HMs for each slice were recorded during application of TBOA. Figure 1a shows representative examples (taken from five HMs) recorded in the presence of TBOA alone (top records; Supplementary Movie 1), or co-applied with nicotine (10 *μ*M) (Figure 1a middle; Supplementary Movie 2), or with carbenoxolone (200 *μ*M) (Figure 1a bottom; Supplementary Movie 3). TBOA induced transients in about 50% of HMs (50±8%, *n*=5 slices), a value reduced to 36±14% (*n*=5 slices) in presence of nicotine, and to 14±7% when carbenoxolone was co-applied (*n*=5 slices). On average, nicotine or carbenoxolone significantly decreased the number of transients evoked by TBOA (Figure 1c; *P*=0.001) as they fell from 4.4±0.4 (*n*=69 HMs) to 2.8±0.2 (*P*<0.05, *n*=40 HMs) in presence of nicotine, or to 2.3±0.3 (*P*<0.05, *n*=18 HMs) with carbenoxolone co-application. However, neither nicotine nor carbenoxolone changed the basal level of [Ca^2+^]_i_ at 10 min. Figure 1d shows a plot of the number of transients against cumulative probability. Thus, the plot for nicotine or carbenoxolone co-application is significantly shifted to the left (*P*≤0.001 for TBOA, solid line *versus* nicotine + TBOA, dotted line; and *P*≤0.001 for TBOA *versus* carbenoxolone + TBOA, dashed line), demonstrating increased probability to observe fewer events than in the presence of TBOA alone.

**Figure 2:**
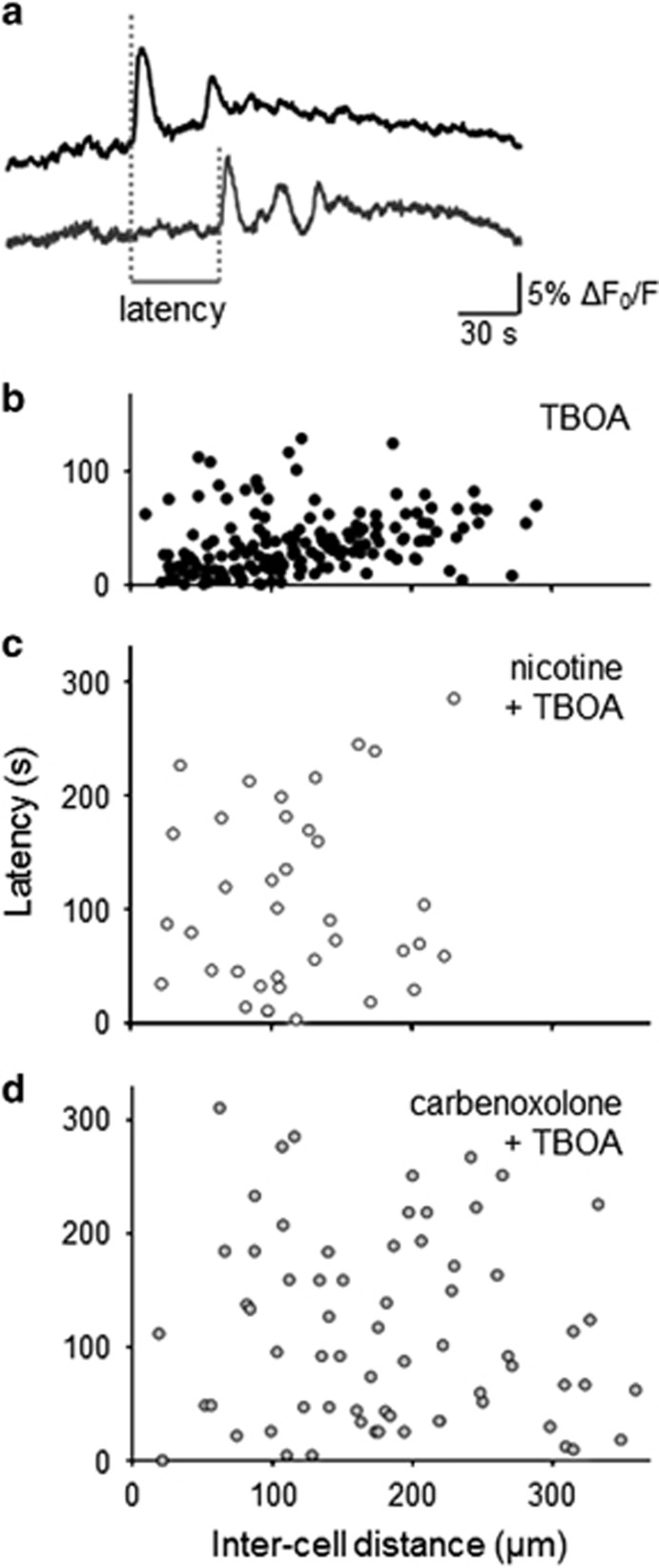
Topographic distribution of Ca^2+^ transients according to inter-cell distance. (**a**) Example of latency analysis taken as interval between Ca^2+^ transient onset in two nearby HMs. (**b**-**d**) Inter-cell latency scatter plots in presence of TBOA (50 *μ*M; (**b**), or nicotine (10 *μ*M) + TBOA (**c**) or carbenoxolone (200 *μ*M) + TBOA (**d**). Each data point represents the latency value calculated for a pair of cells. For data points shown in **b**, a significant linear correlation (*ρ*=0.30, *P*<0.001) between signal latency and cell distance was observed with the analysis of deviance (see Materials and Methods section) in which the regression line for the plot of latency (taken as y) is equal to 21.08 + 0.13 inter-cell distance (taken as x): this result differs in a significant manner (*P*<0.001) from the null linear model, that is, the mean value latency=36.60. Therefore, the regression line appears to be the minimal adequate model to establish that distance has an effect on latency in a statistical sense

As a result of the scattered onset of [Ca^2+^]_i_ transients (Supplementary Movies 1–3), we studied whether topographical distance between two motoneurons was predictive of the closely spaced activity. Thus, Figure 2 compares the inter-neuronal distance with the latency between the first calcium transient of each cell pair as exemplified in Figure 2a in which the long interval between transients indicates lack of coupling. The top plot (Figure 2b), which refers to TBOA-treated cells only, shows how the latency between transients was significantly related to their distance (*ρ*=0.30, *P*<0.001) and that the vast majority of latency values were below 100 s. Conversely, when nicotine or carbenoxolone was co-applied with TBOA (Figures 2c and d), the plots comprised a cloud of widely scattered points with latency values larger than 200 s even for closely located HMs. These data demonstrated the asynchronous occurrence of [Ca^2+^]_i_ changes when nicotine or carbenoxolone were co-applied.

### Nicotine or carbenoxolone modulates Cx36 expression

**Figure 3:**
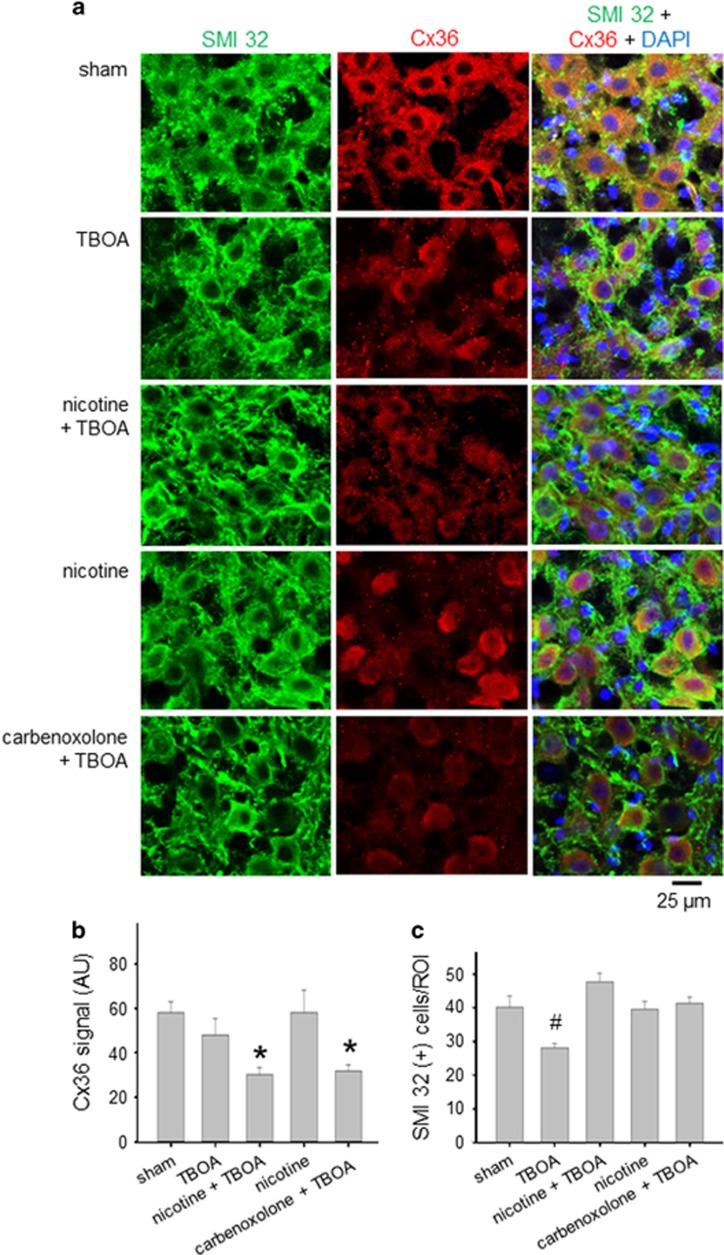
Nicotine and carbenoxolone induce significant reduction in Cx36 expression. (**a**) Example of HMs after 4-h incubation in Krebs (sham), TBOA, nicotine + TBOA, nicotine, or carbenoxolone + TBOA. HMs were labeled with SMI 32 (green pseudocolor; left column), as motoneuronal marker, or Cx36 (red pseudocolor; middle column). The right column shows merged images where DAPI (blue pseudocolor) is used to visualize nuclei. (**b**) Histograms quantify the Cx36 signal decrease after 4h of nicotine + TBOA (Kruskal–Wallis one-way ANOVA: *P*=0.005 among groups; Dunn’s method: *P<0.05 for sham *versus* nicotine + TBOA) or carbenoxolone + TBOA treatments (Dunn’s method: *P<0.05 for sham *versus* carbenoxolone + TBOA). Cx36 mean signal (AU): 58±4.9, sham (*n*=10, *N*=3); 48±7.2, TBOA (*n*=15, *N*=4); 30±3.4, nicotine + TBOA (*n*=14, *N*=3); 58±10, nicotine (*n*=8, *N*=3); 32±2.8, carbenoxolone + TBOA (*n*=11, *N*=4). (**c**) Plot indicates significant HM loss after TBOA treatment, an effect fully prevented by nicotine or carbenoxolone co-application (HMs: 40±3, sham; 28±1, TBOA; 48±3, nicotine + TBOA; 40±2, nicotine; 41±2, carbenoxolone + TBOA; Kruskal–Wallis one-way ANOVA: *P*=0.001 among groups; Holm–Sidak method: ^#^*P*<0.05 for TBOA *versus* sham, nicotine + TBOA, nicotine, or carbenoxolone + TBOA)

As HM bursting is supported by a variety of mechanisms including gap junctions,^15, 16, 26, 27^ the above results raised the hypothesis that nicotine (like carbenoxolone) may impair intercellular communication via Cx36 and Panx1. As mitochondrial dysfunction and motoneuron death are observed after 4-h treatment with TBOA,^13^ we evaluated Cx36 expression (Figures 3a and b; red) in relation to immunoreactivity of single HMs (identified with SMI 32 immunostaining; green). DAPI was used for nuclear staining (blue). In accordance with previous studies,^28, 29^ Cx36 immunoreactivity was distributed through the soma of HMs as shown in Figure 3a (middle column) and quantified in Figure 3b. There was also weak staining of the nucleus in accordance with the report by Belluardo *et al.*^30^ who have detected nuclear Cx36 in rat brain neurons.

In the present experiments, the Cx36 signal was not significantly altered by TBOA alone in the cells that remained after the excitotoxic stimulation, whereas the motoneuronal number after TBOA (Figure 3c) fell by approximately 30%. When nicotine was co-applied with TBOA, the Cx36 immunoreactivity was significantly decreased (Figures 3a and b) and the average number of motoneurons was similar to sham (Figure 3c). An analogous result was observed when carbenoxolone was co-applied with TBOA (Figures 3a and c). Nicotine *per se* did not change the average Cx36 signal (Figure 3a and b) or motoneuron survival (Figure 3c).^13^

**Figure 4:**
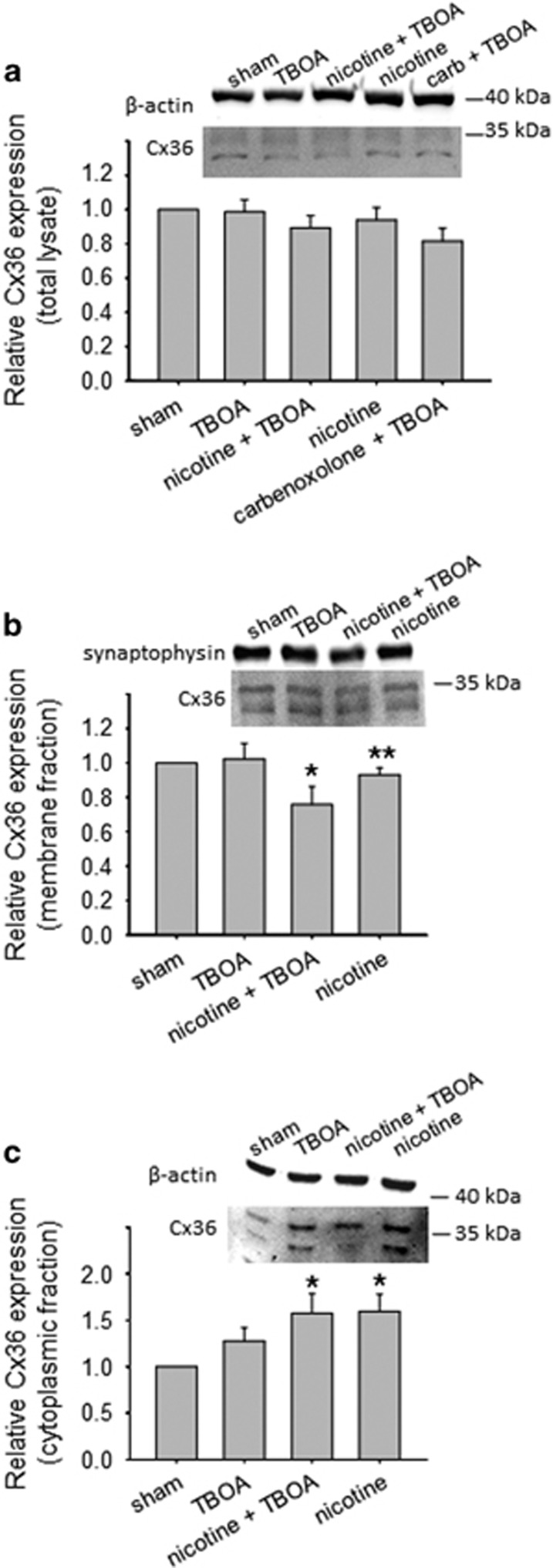
Brainstem Cx36 expression decreases because of nicotine or carbenoxolone application. (**a**) Example of western immunoblotting (top) showing the expression of Cx36 in brainstems incubated for 4 h in Krebs, or treated with TBOA, nicotine + TBOA, nicotine, or carbenoxolone + TBOA. Histograms (bottom) quantifying the significantly decreased Cx36 levels after treatment with nicotine + TBOA (n, *N*=9) or carbenoxolone + TBOA (n, *N*=5; Kruskal–Wallis one-way ANOVA: *P*=0.015 among groups). (**b**and**c**) Example of western blot (top) showing Cx36 expression in membrane (**b**) or the cytoplasmic (**c**) fractions of samples treated as described above. Histograms illustrate how nicotine alone or co-applied induces a significant decrease of Cx36 expression in the membrane pool (**b**, bottom; Kruskal–Wallis one-way ANOVA: *P*=0.069 among groups; Mann–Whitney test: *P=0.016 for sham *versus* nicotine + TBOA and **P=0.008 for sham *versus* nicotine; n, *N*=5) and an increase in the cytoplasmic one (**c**, bottom; Kruskal–Wallis one-way ANOVA: *P*=0.014 among groups; Tukey test: *P<0.05 for sham *versus* nicotine + TBOA, and for sham *versus* nicotine n, *N*=5)

To further investigate the amount of Cx36 expressed under various experimental conditions, western blot experiments were performed as depicted in Figure 4 using brainstem tissue blocks in order to collect a sufficient amount of protein. Even if the nucleus hypoglossus represents only a minor region of this tissue, this approach was earlier useful to detect changes in gene profiling, protein synthesis and expression, and mitochondrial energy production^13, 14^ which, although cannot be directly attributed to HMs (or other brainstem motoneurons), yet they provide an index of the metabolic state of the network and of any ongoing neuroprotective process.

**Figure 5:**
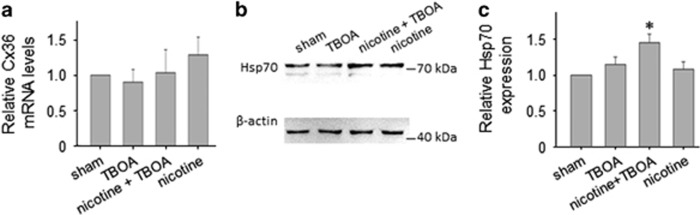
Cx36 mRNA levels and Hsp70 expression. (**a**) Plot illustrating unchanged values of Cx36 mRNA levels among different treatments. Fractional Cx36 expression (AU): 1±0.0, sham; 0.9±0.2, TBOA; 1±0.3, nicotine + TBOA; 1.3±0.2, nicotine (n, *N*=6). (**b**) Example of Hsp70 immunoblotting. (**c**) Histogram quantifying the significant increase of Hsp70 expression after nicotine + TBOA treatment (Kruskal–Wallis one-way ANOVA: *P*=0.027 among groups; Holm–Sidak method: *P<0.05 for sham *versus* nicotine + TBOA; n, *N*=5)

Evaluation of Cx36 total lysate (approximately 36 kDa) samples confirmed unchanged protein quantity after 4-h treatment with TBOA (0.99±0.07, *n*=9 brainstems) or nicotine (0.94±0.07, *n*=6 brainstems; Figure 4a). In support of the immunohistochemical data (Figures 3a and b), we observed that, when samples were treated with nicotine + TBOA or carbenoxolone + TBOA, Cx36 expression was significantly reduced to 0.89±0.07 (*n*=9 brainstems) or 0.82±0.08 (*n*=5 brainstems; *P*=0.015 among groups), respectively. We further explored the distribution of Cx36 in membrane and cytoplasmic compartments. In the case of nicotine exposure, there was a small, yet significant decrease in Cx36 membrane expression (Figure 4b; *P*=0.008 for sham *versus* nicotine; *n*=5 brainstems) concomitant with a corresponding increase in the cytoplasmic fraction (Figure 4c; *P*<0.05 for sham *versus* nicotine; *n*=5 brainstems). The fall in membrane expression of Cx36 was intensified when nicotine was co-applied with TBOA (Figure 4b; *P*=0.016 for sham *versus* nicotine + TBOA; *n*=5 brainstems) in association with a significant rise in the cytoplasmic expression (Figure 4c; *P*<0.05 for sham *versus* nicotine + TBOA; *n*=5 brainstems). Interestingly, no compartmental expression was modified by TBOA alone, perhaps suggesting that the manifestation of the action of this drug required an unchanged Cx36 expression. These effects of Cx36 compartmentalization were not due to changes in its synthesis as qPCR experiments showed no alteration in the Cx36 gene product (Figure 5a; *P*=0.21 among groups; *n*=6 brainstems).

### Hsp70 and AIF contrasting expression in HMs

**Figure 6:**
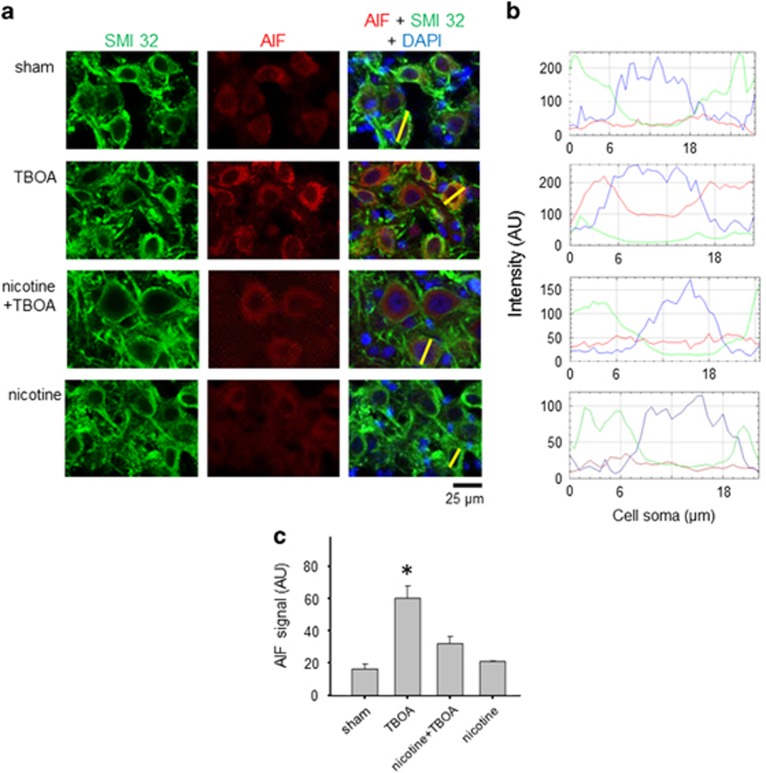
Expression of AIF in HMs. (**a**) Confocal images of SMI 32 (left; green), AIF (middle; red) and DAPI (blue; merged image, right) of motoneurons in control condition or after TBOA (50 *μ*M), nicotine (10 *μ*M) + TBOA, or nicotine alone. (**b**) Line scan analysis for the cells indicated by the bars in **a**. Nuclear area is delimited by the strong DAPI distribution. Note different ordinate calibrations. The large rise in AIF with TBOA is prevented by nicotine. (**c**) Histograms show average AIF intensity signal in cell soma. There is no difference between sham (*n*=9, *N*=4) and nicotine (*n*=6, *N*=3) data. TBOA treatment (*n*=19, *N*=7) induces a large increase in AIF expression (*Kruskal–Wallis one-way ANOVA: *P*<0.001; Dunn’s method: *P*<0.05 for TBOA *versus* sham and TBOA *versus* nicotine). Nicotine + TBOA (*n*=16, *N*=5) elicits a comparatively smaller increase in AIF

In case of excitotoxicity, motoneuronal survival may depend on the relative intracellular expression of Hsp70 and AIF with cell-protecting or cell-damaging properties, respectively.^24, 25^ Figures 5b and c show that, after TBOA or nicotine treatment, Hsp70 expression was unchanged compared with basal sham conditions. When nicotine and TBOA were co-applied, Hsp70 expression was significantly enhanced (*P*=0.027 among groups; *P*<0.05 for sham *versus* nicotine + TBOA; *n*=5 brainstems). Thus, during excitotoxic stimulation, application of nicotine enhanced Hsp70 expression, thereby strengthening the neuroprotective processes of motoneurons. In fact, protection of motoneurons from excitotoxicity depends to a large extent on the binding by Hsp70 of AIF, a mitochondrial factor released by metabolically damaged motoneurons.^24, 25^ When, during excitotoxicity, Hsp70 expression is insufficient to bind AIF, the latter migrates to the cell nucleus and inactivates DNA.^31^ We, therefore, studied immunohistochemical expression of AIF when TBOA was applied alone or together with nicotine. Figure 6a shows examples of AIF immunoreactivity increased after TBOA administration with broad distribution within nuclear (delineated by the blue line in Figure 6b for DAPI staining) and non-nuclear compartments as indicated by the line scan red trace that had higher value throughout (Figure 6b; note different ordinate scale for the various treatment protocols). Vice versa, the level of AIF was low in the presence of nicotine with or without TBOA (Figure 6b), suggesting that nicotine kept the AIF level at sham-like condition. These data are quantified in the bar graph of Figure 6c in which the average fluorescence signal (AU) of AIF in basal condition was 16.1±3.2 (*n*=9 slices) and significantly increased to 60±7.8 (*P*<0.05, *n*=19 slices) after TBOA exposure, whereas it was low when nicotine was co-applied with TBOA (*n*=16 slices). It should be noted that nicotine (*n*=6 slices) *per se* left unchanged the AIF immunoreactivity compared with sham.

### Unchanged expression of Panx1

**Figure 7:**
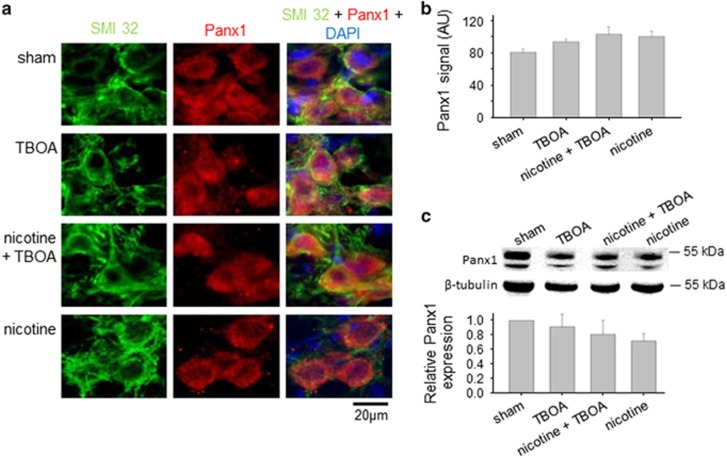
Panx1 expression remains unchanged. (**a**) Example of HMs after 4-h incubation in Krebs (sham), TBOA, nicotine + TBOA, or nicotine, labeled with the neuronal marker SMI 32 (left column; green), or Panx1 (middle column; red). Merged images are shown on the right column where DAPI (blue) is used as nuclear marker. (**b**) Histograms quantifying unchanged levels of Panx1 among the treatments described above. (**c**) Example of western immunoblotting (top) showing the unchanged expression of Panx1 in brainstems incubated as described above

We sought to understand whether nicotine or carbenoxolone could change other proteins such as Panx1 reputed to make gap junctions. To this end, immunohistochemical and western blot experiments were performed as illustrated in Figures 7a and c. Hence, Panx1 immunoreactivity was readily detected in HMs as depicted in Figure 7a and remained unchanged following TBOA with or without nicotine protocols (Figure 7b). Similarly, Panx1 protein expression was very similar among all these treatments, indicating that Panx1 was not involved in nicotine neuroprotection at least within the 4-h experimental timeframe.

## Discussion

The novel finding of this study was the demonstration that, during an excitotoxic stimulus, nicotine perturbed the emergence of coordinated Ca^2+^ transients, decreased the expression of Cx36 at membrane level, enhanced the expression of Hsp70 while diminishing the one of AIF. These data were critical components of the neuroprotective action by this alkaloid. As many effects of nicotine were replicated by carbenoxolone, it is likely that inhibition of Cx36 was essential to uncouple motoneurons from their collective bursting behavior that was prodromic to cell distress and death.

### Motoneurons wired together, die together

Inhibition of glutamate uptake recruits clusters of motoneurons in group bursting that, if it continues unabated, will lead to significant neuronal death.^12^ As our recent work indicates that nicotine largely suppresses bursting,^13^ we investigated how this phenomenon was expressed within the HM population (recorded as Ca^2+^ transients) and its spatio-temporal correlation. Neighboring HMs often generated time-correlated Ca^2+^ transients suggesting their functional ensemble. It has been demonstrated that carbenoxolone acting as a pharmacological blocker of gap junctions, inhibits motoneuron synchronization in vertebrates and invertebrates.^32, 33^ Thus, following application of carbenoxolone as well as nicotine, bursting became sparse and, importantly, this event was followed by significant protection of HMs from death. This realization implies that the severity of damage was perhaps dependent on the collective activation and recruitment of clusters of HMs into pathological discharges, and led us to study the role of gap junctions (known to exist among HMs; 15,16) mediated by Cx36 in this process.

Even if carbenoxolone is not a very specific blocker of gap junctions, the salient contribution by these structures to the collective bursting behavior and their inhibition by nicotine were validated with immunohistochemistry data as shown in Figures 3a and b.

### Connexins and cell death

Cx36 is the most prevalent gap junction protein expressed by neurons.^17, 18^ It electrically couples neighboring cells by allowing transcellular communication and exchange of Ca^2+^, Na^+^, K^+^, and small (<1–1.5 kDa) hydrophilic molecules.^18, 34^ Many factors regulate the activity of gap junction channels, including changes in voltage, [Ca^2+^]_i_, pH,^35^ connexin phosphorylation^35, 36^ and ROS.^37^ In the past few years, many other components of intra/extracellular signaling have been associated with plasma membrane hemichannels such as facilitation of glutamate release from astrocytes^38^ and apoptosis.^34^ Indeed, gap junction-related apoptosis has been demonstrated by carbenoxolone-mediated prevention,^39^ and by the spreading of death signals within a cell cluster via gap junctions.^39, 40^ This mode of propagation from injured to uninjured close neighbor cells is classified as bystander killing (the ‘kiss of death’).^17, 34^ An interaction among connexins and mitochondrial AIF in cardiomyocytes has been described with potential effects on mitochondrial respiration and ROS signaling.^41^ Whether this process could also occur in motoneurons remained unclear.

During NMDA receptor-mediated excitotoxicity, a strong reduction in neuronal death of cultured cortical neurons is obtained when Cx36 is pharmacologically blocked or genetically ablated, indicating that the expression level of Cx36 critically modulates neuronal death.^27, 42^ Thus, the observation of basal expression of Cx36 by HMs made this protein a likely candidate in the excitotoxic cell death. It was of interest that, following glutamate uptake block and loss of a number of HMs, surviving cells did not show impaired Cx36 expression, alluding to the possibility that these cells had relied on certain intrinsic mechanisms to withstand the injury process.

### Nicotine prevents HM excitotoxic death via Cx36 downregulation

In endothelial cell cultures, nicotine, via membrane ACh receptors, downregulates various connexins through intracellular pathways^21, 22^ that impact their turnover.^22^ In excitotoxic stress, we actually detected a fall in Cx36 immunoreactivity of HMs when nicotine (or carbenoxolone) was applied with TBOA, a result that was accompanied by no significant loss of HMs. Although nicotine can decrease excitatory synaptic transmission on HMs,^13, 20^ the analogy with the effects by carbenoxolone and the downregulated immunopositivity of Cx36 suggested that Cx36 were important targets to restrain excitotoxicity. Previous studies have shown that the turnover of Cx36 is rapid (3 h),^43^ a result consistent with fast changes in protein expression without changes in Cx36 synthesis as shown by the negative qPCR data of the present experiments. Previous studies have demonstrated an early decrease in Cx43 expression by carbenoxolone.^44^ Thus, it is feasible that blocking Cx36 directly with carbenoxolone or via intracellular signaling with nicotine led to decreased immunosignal of this protein at HM level because degradation was enhanced and/or replenishment impaired. In the case of nicotine, one parsimonious hypothesis is that this drug delayed Cx36 transportation from intracellular stores to the plasma membrane so that ongoing Cx36 degradation could not be efficiently compensated. Our view is consistent with the notion of nicotine uncoupled cells via increased proteolysis of connexins.^22^

Circumstantial evidence was sought with tissue fractionation experiments in which we sought the relative distribution of Cx36 among membrane and cytosolic fractions. Despite the limitation inherent in the use of brainstem tissue blocks containing a heterogeneous cell population, we detected a significant decrease in the Cx36 membrane fraction together with a notable rise in its cytoplasmic fraction that also comprises contributions from intracellular stores. These data implied that the expression and perhaps the function of Cx36 at membrane level were impaired with nicotine application. Although the present data cannot, of course, be attributed to discrete changes in HMs alone because of the use of brainstem tissue, they allowed us to point to Cx36 as an important target for nicotine neuroprotection: should this process extend to other brainstem neurons (or glia), it may reinforce the widespread impact of nicotine effect against excitotoxicity. Our model also indicated that the effect by nicotine had a degree of specificity as Panx1 expression was unchanged by nicotine. We cannot, however, rule out that the long life-cycle of Panx1^45^ had precluded detecting a later alteration following nicotine application.

### Nicotine modulated Hsp70 expression as a gateway to cell survival against AIF-mediated death

One further result of interest was the observation that nicotine and TBOA application evoked a significant increase in Hsp70 expression, whereas nicotine or TBOA alone did not change this protein. As the intracellular expression of Hsp70 is an important biomarker of the ability of motoneurons to resist to excitotoxicity,^24^ we surmise that surviving cells had an adequate Hsp70 expression although these data were obtained from brainstem tissue rather than single HMs. The perturbation triggered by TBOA plus the effects of nicotine probably synergized to raise Hsp70 expression and extend neuroprotection. These observations, therefore, provided an interesting clue to further explore the mechanism of nicotine neuroprotection. Our view was in line with reports of nicotine ability to enhance Hsp70 expression in lung vessels and ovarian tissue culture,^46, 47^ and to block AIF lethal translocation into the nucleus.^31^ The immunofluorescent data showed that, at HM level, TBOA clearly enhanced the expression of AIF, a factor released by distressed mitochondria, and that this biomarker was distributed throughout the motoneuron including its nucleus. Co-application of TBOA and nicotine normalized the rise in AIF expression and restituted sham-like condition, suggesting that the effect of this death factor was inhibited likely as a consequence of the raised Hsp70 expression in the cytoplasm. These results indicate that neuroprotection by nicotine was, at least *in vitro*, more systematic than the effect produced for example by pharmacological depression of glutamate release with riluzole.^11^ We propose that the additional target of Cx36 uncoupling by nicotine is important to confer prevention of excitotoxicity.

### A scenario for motoneuron protection by nicotine

**Figure 8:**
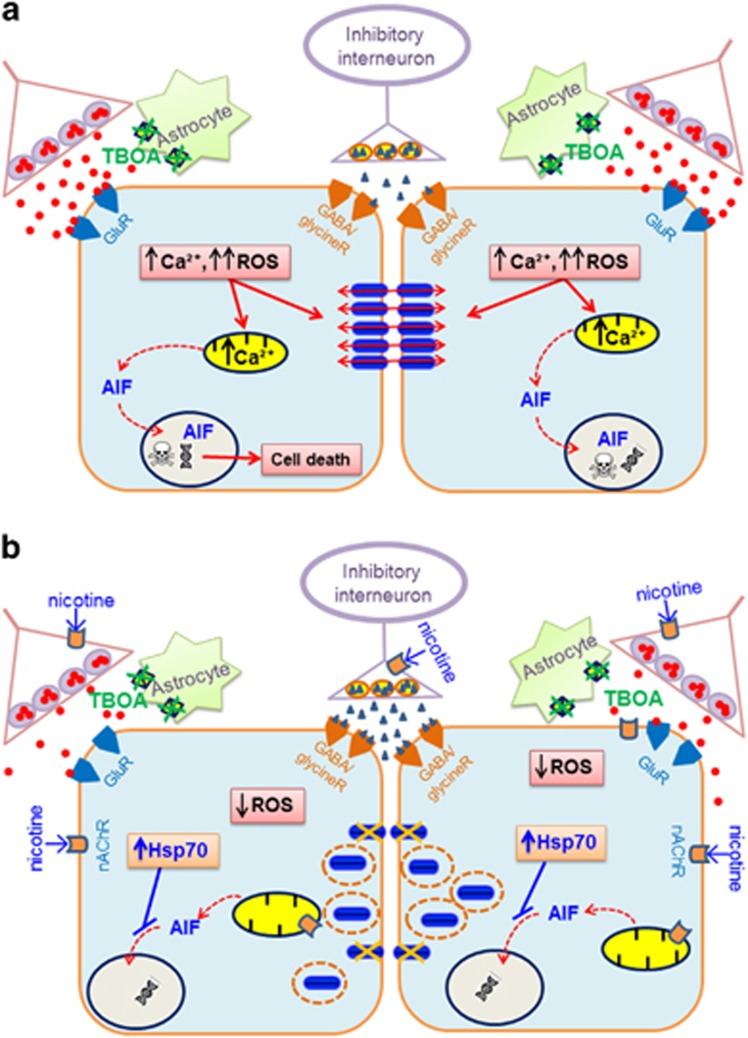
Idealized diagram to account for the toxic effects by TBOA and their antagonism by nicotine on HMs. (**a**) In the presence of TBOA, glutamate (filled red circles) released from presynaptic terminals acts on glutamatergic receptors to depolarize HMs. TBOA-mediated inhibition of glutamate uptake by astrocytes leads to intensification of glutamate effects with build-up of intracellular free Ca^2+^, which acts on mitochondria and favors production of ROS, release of AIF and DNA damage. This process is greatly amplified by the bidirectional (red arrows) operation of gap junctions (Cx36; filled blue cylinders) that spread and recruit HMs into a hyperexcitable state with deleterious consequences on cell survival. (**b**) In the presence of nicotine, the neurotoxic effect of TBOA is largely attenuated. Nicotine exerts multiple effects via nAChRs located on presynaptical terminals (with consequent decrease in glutamate release, and increase in the inhibitory response), HM membrane and even mitochondria. Through these effects, nicotine strongly decreases the motoneuron coupling via gap junctions through redistribution of Cx36 subunits, therefore disjoining motoneurons from their collective excitatory behavior. Our hypothesis is that this process is accompanied by inhibition of Cx36 activity, reduction in production of ROS and facilitation of the effect of Hsp70 to sequester AIF and prevent its nuclear migration. For references, see text

Figure 8 depicts an idealized diagram to account for the process of HM death evoked by glutamate uptake block and the intervention levels exerted by nicotine. Excitotoxicity, induced by block of excitatory amino-acid transporters,^48^ severely damages motoneurons by excessive glutamate receptor stimulation that induces strong network bursting^11, 12, 13, 26^ among electrically coupled HMs (Figure 8a). This phenomenon elicits excessive Ca^2+^ influx, which depolarizes the neuronal membrane potential to activate further Ca^2+^ influx^49, 50^ and intracellular second messengers in a death cell cascade.^51, 52^ The role by mitochondria in Ca^2+^ buffering may lead to mitochondrial membrane depolarization^49, 50, 53^ and perturbation of the respiratory chain.^49, 50^ This process is compounded by ROS-evoked oxidative stress.^13, 54, 55^ In this vicious sequence, cell damage is extended via gap junctions^17, 27, 56^ activated by ROS.^37^

To contrast excitotoxic damage, a potential device is activation of nAChRs expressed on interneurons and HMs.^13, 14, 20, 57^ nAChRs operate through a cycle of activation, desensitization and reactivation^58^ that, in the case of brainstem synaptic transmission with diffuse inputs impinging on HMs, include early facilitation of glutamate release followed by depression, whereas synaptic inhibition is enhanced^13, 20^ (Figure 8b). Direct activation of nAChRs on motoneurons reduces ROS production, enhances Hsp70 expression and uncouples Cx36-mediated intercellular communication. The mechanism linking nAChR activation to inhibition of Cx36 activity will require future investigation. We can posit that, as nicotine enhances protein kinase C (PKC),^59, 60, 61^ PKC-mediated connexin phosphorylation is a putative process to reduce gap junction-dependent communication.^62^ Finally, our data do not intend to support a neuroprotecting role of smoking, rather to prompt a strategy for further investigation of inhibiting motoneuron disease *in vitro* and *in vivo* with novel tools to stimulate nAChRs.

## Materials and methods

### Ethical approval

All experiments were performed following the ethical guidelines for the use and the care of laboratory animals of National Institutes of Health. The Scuola Internazionale Superiore di Studi Avanzati (SISSA) ethics committee (prot. 3599, 28 May 2012) approved all treatment protocols, which were in agreement with the European Union rules for animal experimentation. We made all the effort to minimize the use and the suffering of the animals, and reduce their number for experimentation.

### Slice preparation and drug application protocols

Experiments were carried out on neonatal Wistar rats (postnatal days 1–6; P1–P6), rapidly decapitated under i.p. urethane anesthesia (10% solution, 0.1 ml injection volume). Brainstems were removed in continuously carbogenated (95% O_2_ and 5% CO_2_) ice-cold Krebs solution containing (in mM): 130 NaCl, 3 KCl, 1.5 NaH_2_PO_4_, 1 CaCl_2_, 5 MgCl_2_, 25 NaHCO_3_ and 18.5 glucose (pH 7.4; 300–330 mOsm/l). For Ca^2+^ imaging and immunohistochemical experiments, slices (270–450 *μ*m thick) containing the nucleus hypoglossus were immediately cut with a vibrating tissue slicer (Leica VT 1000S, Wetzlar, Germany). Slices (or intact brainstems for molecular biology) were then rapidly transferred to an incubation chamber for 20 min at 32°C and then recovered for 10 min at room temperature. Details of the experimental procedure were previously published.^12, 63^

With the exception of slices for the calcium-imaging technique, samples were subsequently incubated for 4 h at room temperature in continuously carbogenated Krebs solution (sham), TBOA (50 *μ*M; Sigma-Aldrich, St. Louis, MO, USA), TBOA + nicotine (10 *μ*M; Sigma-Aldrich), nicotine, or TBOA + carbenoxolone (200 *μ*M; Sigma-Aldrich) and processed as indicated later. Experiments were run in parallel to minimize bias.

### Intracellular Ca^2+^ imaging [Ca^2+^]_i_

In accordance with formerly described protocols,^12, 64^ slices (270 *μ*m thick) were loaded with the fluorescent Ca^2+^ dye Fluo-3 AM (4 *μ*M, Molecular Probes, Invitrogen, Carlsbad, CA, USA) for 1 h in continuously carbogenated Krebs solution. After 30-min wash, samples were transferred into the recording chamber of the Nikon Eclipse T1 microscope (Nikon, Tokyo, Japan), where the nuclei hypoglossi were identified with a 40x objective (aperture 0.60). Drug concentrations were chosen on the basis of previous reports^12, 13^ and applied acutely in accordance with the following protocols (10 min): TBOA (50 *μ*M), nicotine (10 *μ*M) + TBOA (50 *μ*M); carbenoxolone (200 *μ*M) + TBOA (50 *μ*M). Ca^2+^ fluorescent emission was excited at a fixed wavelength of 488 nm generated by a Nikon intensilight C-HGFI lamp (Nikon) and detected with the digital CMOS camera ORCA-Flash 4.0 (Hamamatsu Photonic, Hamamatsu City, Japan). Images were acquired with the Fiji software (ImageJ, Wayne Rasband, National Institute of Health, Bethesda, MD, USA)^65^ with 150 ms exposure time. In each slice, a small region of interest (ROI) was placed over about 30 randomly distributed motoneurons easily recognizable for their somatic diameter (> 20 *μ*m). Traces extrapolated with Igor Pro software (version 6.37, Wavemetrics, Lake Oswego, OR, USA) were analyzed with the software Clampfit 10.0 (Molecular Devices Corporation, Sunnyvale, CA, USA). Ca^2+^ transients were analyzed if events had a duration <20 s and a rise phase faster than the decay. Transients were expressed as ΔF/F_0_, the amplitude fractional increase, where ΔF is the fluorescence rise over baseline, and F_0_ the baseline fluorescence level; [Ca^2+^]_i_ elevations were considered significant when they exceeded five times the noise S.D.^66, 67^

### Immunohistochemistry

At the end of 4-h experiments, slices were fixed in PBS containing 4% paraformaldehyde for 4 h at 4 °C, treated for cryoprotection in 30% sucrose for 72 h at 4 °C, and finally frozen for at least 12 h in an embedding mounting medium. Embedded slices were then cut with a cryostat in 30 *μ*m tissue sections. Samples were blocked (in a PBS-based solution containing: 10% normal goat serum, 50% BSA and 3% Triton X-100) for 2 h at room temperature and incubated overnight at 4 °C with the primary antibodies anti-SMI 32 (mouse monoclonal, 1:200 dilution; cat. #: 801701; BioLegend, San Diego, CA, USA; for validation see Nani *et al.*^63^), anti-Cx36 (rabbit polyclonal, 1:300 dilution; cat. #: ACC-209; Alomone, Jerusalem, Israel),^29^ anti-Panx1 (rabbit polyclonal, 1:300 dilution; cat. #: ACC-234; Alomone)^68^ or anti-AIF (rabbit polyclonal, 1:200 dilution; cat. #: AB16501; Millipore, Billerica, MA, USA).^69^ AlexaFluor 488 and 594 (1:500 dilution; Life Technologies, Carlsbad, CA, USA) were used as secondary antibodies and applied for 2 h at room temperature. Antibodies were diluted in an antibody PBS-based solution containing: 2% normal goat serum, 10% BSA and 1% Triton X-100. After secondary antibody incubation, slices were rinsed and stained with the DNA dye DAPI diluted in PBS (1:1 000; Sigma-Aldrich), for 20 min at room temperature. To reduce fading, slices were mounted with fluorescence mounting medium (Dako, Glostrup, Denmark) and images acquired by either a Zeiss Axioskop2 microscope (20x; Oberkochen, Germany) or a confocal Nikon microscope (40x in oil) with 1 *μ*m z sectioning. Images were analyzed with the Volocity software (PerkinElmer, Waltham, MA, USA).

### Western blot

Analysis of quantitative and qualitative protein expression was performed with a standard western blot technique on whole brainstem treated as described above in accordance with the previously published protocol.^70^ Total lysates, cytoplasmic and membrane fractions were analyzed. Total lysates were obtained by homogenizing samples in CHAPS buffer solution (0.5% CHAPS, 50 mM Tris pH 7.5, 1 mM EDTA, 150 mM NaCl, 10% glycerol plus protease inhibitors mixture and reducing agents; Complete, Roche Applied Science, Basel, Switzerland; and Sigma-Aldrich). A hypotonic lysis buffer (10 mM HEPES pH 7.9 with 1.5 mM MgCl_2_ and 10 mM KCl plus protease inhibitors mixture and reducing agents) was used to process samples for membrane and cytoplasmic extraction, which were then centrifuged for 5 min at 4000 r.p.m. at 4 °C and the supernatant transferred to ultracentrifuge tubes for centrifugation (1 h) at 100 000 *g*. Cytoplasmic proteins were considered those in the supernant, whereas the pellet was formed by membrane proteins, which were re-suspended in the extraction buffer (20 mM HEPES pH 7.9, with 1.5 mM MgCl_2_, 0.2 mM EDTA, 25% glycerol, 1% SDS plus protease inhibitors mixture and reducing agents). Samples were then immunoblotted with rabbit anti-Cx36 (1:200, Alomone), rabbit anti-Panx1 (1:400, Alomone), mouse anti-Hsp70 (1:5000, Abcam, Cambridge, UK), mouse anti-*β*-actin (1:2000, Sigma-Aldrich), mouse anti-*β*-tubulin (1:2000, Sigma-Aldrich), or mouse anti-synaptophysin (1:10 000, Millipore) antibodies. As both Cx36 and Panx1 encode consensus sites for phosphorylation and glycosilation, it is, thus, common to observe more than one band in the immunoblotting gels^45, 71^ as reported in this study as well. The enhanced chemiluminescence light system (ECL, Amersham Bioscience, Piscataway, NJ, USA) was used to detect signals recorded with the digital imaging system Alliance 4.7 (UVItech, Cambridge, UK) and quantified with the software Alliance LD2-77-WL (UVItec).

### Real-time PCR

PCR experiments were performed on total RNA isolated from tissues, treated as described above, using the Triazol reagent (Invitrogen). RNase-free DNase (Ambion, Austin, TX, USA) was used for RNA extraction and cDNA was purified using the RNeasy Mini Kit (Qiagen, Hilden, Germany), according to the respective manufacturer’s instructions. Single-strand cDNA samples were obtained using the iScriptcDNA Synthesis Kit (Bio-Rad, Hercules, CA, USA) from at least 20 ng of purified RNA. To determine mRNA expression, the following primers were used: (1) Cx36: forward, 5′-ATACAGGTGTGAATGAGGGAGGATG-3′, reverse, 5′-TGGAGGGTGTTACAGATGAAAGAGG-3′^72^ (2) Actb: forward, 5′-GTGGGGCGCCCCGGCACCA-3′, reverse, 5′-CTCCTTAATGTCACGCACGATTT-3′^73^ (3) Hprt: forward, 5′-TCCTCATGGACTGATTATGGACA-3′, reverse, 5′-TAATCCAGCAGGTCAGCAAAGA-3′^74^ (4) Rpl13A: forward, 5′-TCCTCATGGACTGATTATGGACA-3′, reverse, 5′-TAATCCAGCAGGTCAGCAAAGA-3′.^74^ The new synthesized cDNA was amplified using the oligonucleotide primer listed above, the nucleic acid stain iQ SYBER Green Supermix (Bio-Rad) and an iCycler IQ Real-time PCR System (Bio-Rad).

### Statistics

Results were expressed as means±S.E.M. and collected from at least three different experiments, where *n* refers to the number of slices or brainstems for each independent experiment, as indicated, and *N* refers to the number of times an experiment was repeated. Using the standard software SigmaStat 3.5 (Systat Software, Inc., Chicago, IL, USA), we first run an ANOVA test and the normality test to discriminate between parametric and non-parametric data. Subsequently, as directed by the software, the Mann–Whitney test was used for comparing two non-parametric groups. Multiple groups were first analyzed with the Kruskal–Wallis one-way ANOVA on ranks when data were non-parametric and further analysis was carried out with a software directed test (Dunn’s method, Holm–Sidak method or Tukey test). The use of each test for data analysis is indicated in the corresponding Figure legends. Cumulative probabilities were compared with the Kolmogorov–Smirnov test. To analyze the relation between Ca^2+^ transient latency and cell topographical distribution, we applied the linear correlation method based on the analysis of deviance.^75^ In this way, we observed that that the regression line latency for the experiments with TBOA alone differed in a significant manner (*P*<0.001) from the null linear model, that is, the mean value latency=36.60. Thus, the regression line appears to be the minimal adequate model.^76^ Groups of data were accepted as statistically different if *P*≤0.05.

## Supplementary Material

Supplementary Movie Titles
