# Supplementary material for: Nicotine protects rat hypoglossal motoneurons from excitotoxic death via downregulation of connexin 36
Source: Cell Death Dis. 2017 Jun 15;8(6):e2881–. doi: 10.1038/cddis.2017.232 (PMC5520892; doi:10.1038/cddis.2017.232)
Supplement: Supplementary Movie Titles [file cddis2017232x1.docx]

**Movie 1.** TBOA application onto hypoglossal motoneurons**.**

**Movie 2.** Nicotine + TBOA application onto hypoglossal motoneurons.

**Movie 3.** Carbenoxolone + TBOA application onto hypoglossal motoneurons
